# Bedside cerebral microvascular imaging of patients with disorders of consciousness: a feasibility study

**DOI:** 10.3389/fnins.2025.1518023

**Published:** 2025-02-12

**Authors:** Qianqian Ge, Lijie Huang, Qiang Fu, Shuai Han, Rui Wang, Jianghong He, Changhui Li, Jianwen Luo, Long Xu

**Affiliations:** ^1^Deparment of Neurosurgery, Beijing Tiantan Hospital, Capital Medical University, Beijing, China; ^2^School of Biomedical Engineering, Tsinghua University, Beijing, China; ^3^Department of Biomedical Engineering, College of Future Technology, Peking University, Beijing, China; ^4^National Biomedical Imaging Center, Peking University, Beijing, China

**Keywords:** disorders of consciousness, ultrafast power Doppler imaging, craniectomy, cerebral blood flow, bedside

## Abstract

**Background:**

Efficient bedside neurofunctional monitoring is crucial for managing disorders of consciousness (DoC). Ultrafast Power Doppler Imaging (uPDI) outperforms traditional Ultrasound in bedside for assessing the microcirculatory system. However, intracranial blood flow imaging traditionally faces limitations due to the skull’s impedance. This constraint is circumvented in common post-craniectomy DoC patients, who present a unique observational window for uPDI.

**Methods:**

We conducted uPDI scans on five DoC patients of different ages and consciousness levels who had undergone decompressive craniectomy. We compared the imaging results from uPDI with traditional PDI and identified the physiological and pathological conditions with uPDI.

**Results:**

Detailed microvascular images of both cortical and subcortical areas were obtained using uPDI through the craniectomy window. Notably, uPDI demonstrates high sensitivity and imaging depth, revealing microvessels as small as 320 μm in diameter at 4 cm depth, and detecting blood flow signals up to 6 cm beneath the scalp.

**Conclusion:**

Through the decompressive cranial windows of DoC patients, we obtained cerebral microvascular images with significantly higher sensitivity without the need for contrast agents.

**Significance:**

Our research provides a novel bedside cerebral microcirculation imaging method for patients with DoC, offering convenient neurofunctional assessment to improve the clinical management of DoC patients.

## Introduction

Disorders of consciousness (DoC), such as coma, vegetative state/unresponsive wakefulness syndrome (VS/UWS), minimally conscious state minus (MCS-), and minimally conscious state plus (MCS+), arise from severe brain injuries (Thibaut et al., 2020; Thibaut et al., 2019). These conditions make clinical assessments extremely challenging due to patients’ inability to communicate, necessitating the use of neuroimaging and electrophysiological tools for diagnosis and treatment decisions. Although technological advances in magnetic resonance imaging (MRI) have provided high-resolution images for structural and functional analysis, the special environmental and operational demands exclude its bedside applicability for DoC patients (Fischer et al., 2022; Song et al., 2020). Bedside neural assessment modalities like electroencephalography (EEG) and functional near-infrared spectroscopy (fNIRS) offer practical alternatives but are hindered by lower spatial resolution and penetration depth. Despite adopting particular electrode or source-detector placements, these modalities mainly capture cortical activity and usually overlook subcortical regions crucial for the recovery of consciousness (Ferrari and Quaresima, 2012; Molteni et al., 2023; Si et al., 2023; Song et al., 2020). Therefore, the development of non-invasive, bedside, continuous monitoring systems that can probe deeper brain functions is essential for real-time assessment and could significantly influence the clinical management of DoC patients.

Clinical Ultrasound (US) imaging has been widely used because it is non-invasive, ionizing-free, real-time, cost-effective, and capable of performing bedside imaging. Among those, color Doppler imaging (CDI) and power Doppler imaging (PDI) have been commonly adopted for the assessment of vessels (Saito et al., 2014). However, the sensitivity of traditional PDI to small vessels is low due to the short Doppler ensemble length limited by focused imaging with low frame rate. In the past decade, with the development of ultrasound plane wave imaging at ultra-high frame rate (more than 1 kHz) (Montaldo et al., 2009), as well as advanced image reconstruction algorithms (Demene et al., 2015), the ultrafast power Doppler imaging (uPDI) is developed with significantly improved sensitivity to low-speed blood flow in small vessels over traditional PDI (Huang et al., 2023b; Huang et al., 2022; Stanziola et al., 2018; Wang et al., 2023) (Table 1). uPDI performs plane wave imaging at ultrahigh frame rate, which enables using longer Doppler ensemble length and advanced spatio-temporal clutter filters like singular value decomposition (SVD) to separate low-speed blood flow signals from background tissue signals better than traditional PDI. Therefore, uPDI can visualize microvessels (at the scale of several hundred μm) without contrast agents, and has great potential for monitoring neuro-related hemodynamics in small vessels in the brain.

**Table 1:** Comparison between traditional PDI and uPDI.

Modality	Traditional PDI	Ultrafast PDI (uPDI)
Frame rate of data acquisition	~30 Hz	>1,000 Hz
Ensemble length	~10	~100
Clutter filter	High-pass filter	Singular value decomposition
Flow speed sensitivity	~cm/s	~mm/s
Minimal detectable vascular size	~mm	~100 μm

However, the application of ultrasound in neuroimaging is limited primarily due to the barrier posed by the thick skull. A few exception cases to use uPDI for the imaging of the central nervous system include implementation intraoperatively (Huang et al., 2021; Xu et al., 2023) or through the unclosed front fontanel structure of healthy full-term neonates (Xu et al., 2023), preterm neonates (Barletta et al., 2021; Huang et al., 2023a), and neonates with brain injury (Hwang et al., 2022; Xu et al., 2023). However, uPDI has not been used for cerebral microvascular imaging of DoC patients.

**Figure 1:**
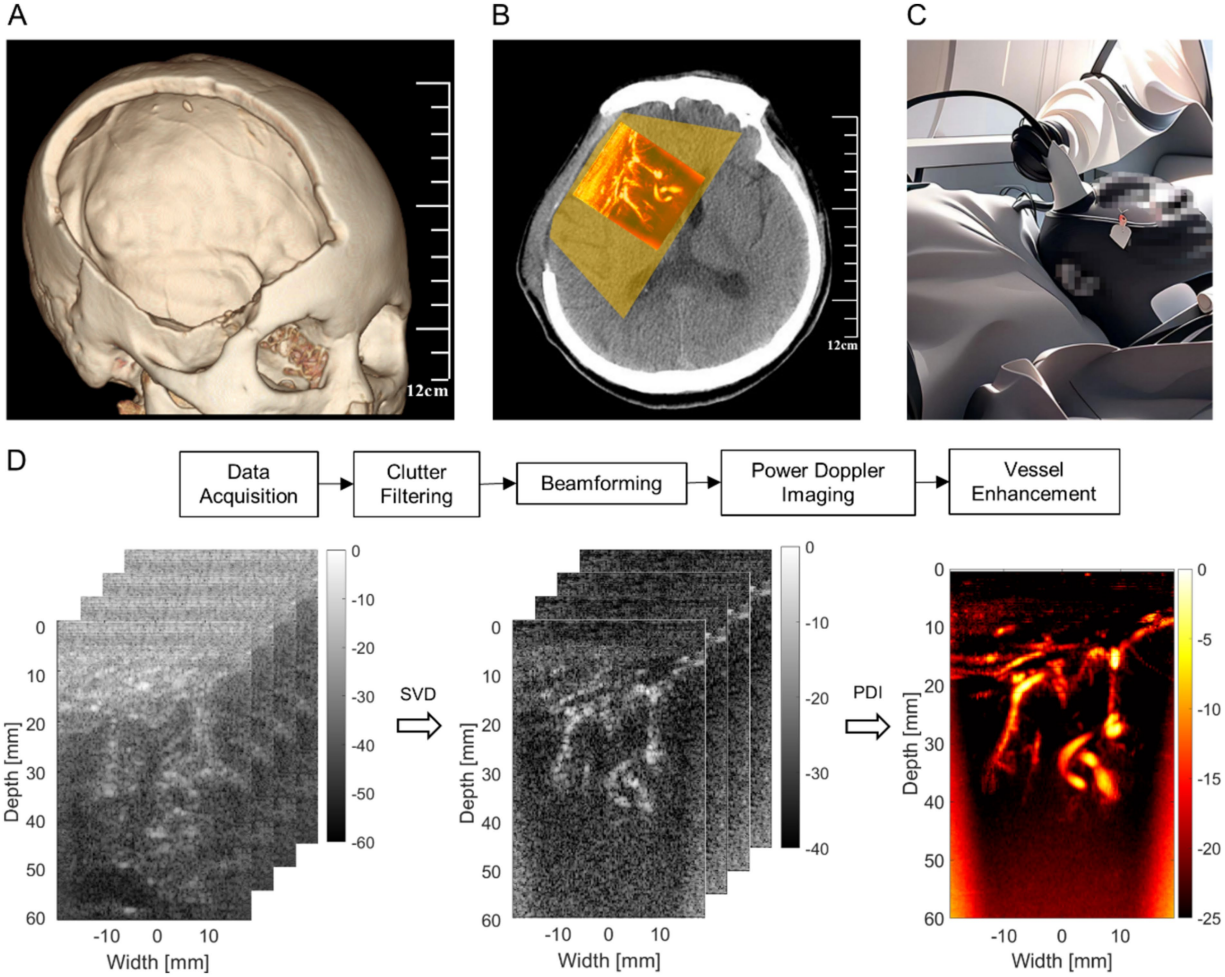
Technical illustration. **(A)** The range of standard decompressive craniectomy. **(B)** The observable range and example of uPDI through decompressive craniectomy window. **(C)** Bedside ultrasound monitoring. **(D)** Ultrafast power Doppler image processing workflow.

The skull barrier for US can be particularly bypassed for certain DoC patients since neurosurgeons often temporally remove part of their skulls (Zaed et al., 2022). In the acute phase of the severe brain injury leading to DoC, there is usually an increase in intracranial contents (such as cerebral hemorrhage) combined with brain tissue swelling, which collectively results in significantly elevated intracranial pressure. To prevent life-threatening brain herniation, neurosurgeons often urgently perform decompressive craniectomy to reduce intracranial pressure by removing a bone flap (Risdall and Menon, 2011). If the decompressive craniectomy window is too small, the elevated intracranial pressure can push the brain tissue out of the window, causing secondary damage. Therefore, the bone flap removed during the decompressive craniectomy is usually of a relatively large size, covering the frontal, temporal, and parietal regions on one or both sides (Figure 1A) (Michel et al., 2009). The removed bone flap by craniectomy operation is typically repaired after 2–3 months when the patient’s condition has stabilized (Zaed et al., 2022). This provides uPDI with a time window to observe changes in cerebral blood flow and brain activities in multiple regions (Figure 1B). Moreover, this time window is also when patients mostly require convenient and timely bedside monitoring of neurological function (Figure 1C).

In this study, for the first time, we explored uPDI to non-invasively scan the brain of five DoC patients with the skull flap removed and compare the imaging results with traditional PDI. Then, we evaluated the performance of uPDI and discussed the limitations and prospects of uPDI in practical clinical applications for DoC patients.

## Methods

### Participated patients

We enrolled five patients with DoC, who underwent decompressive craniectomy before and had not yet undergone cranioplasty, from October 2022 to May 2023 in Beijing Tiantan Hospital. All enrolled patients had stable conditions, without unstable intracranial pressure, uncontrolled hydrocephalus, acute cerebral infarction, cerebral hemorrhage, or other complications. The level of consciousness in patients was assessed using the Coma Recovery Scale-Revised (CRS-R) (Giacino et al., 2004), and the patient prognosis was evaluated through a follow-up visit 3 months after treatment in our hospital. The overall research protocol was approved by the Ethics Committee of Beijing Tiantan Hospital (No. KYSQ 2021–396-01). Informed consent was obtained from the legal representatives of the subjects. To be noticed, although uPDI revealed either abundant or scarce blood flow signals, as this is a novel technique that had not been strictly verified, these results did not affect the clinical diagnosis and treatment decisions for the patients.

### Data acquisition

Ultrafast ultrasound channel radio-frequency (RF) data were acquired using a programmable ultrasound platform (Vantage 256, Verasonics, Kirkland, WA, USA), equipped with an L13-4 linear array probe with a pitch of 0.3 mm. The center transmit frequency was 6 MHz and a five-angle plane wave compounding (evenly distributed between −6° and 6°) was used at the pulse repetition frequency of 5,000 Hz, leading to an effective frame rate of 800 Hz after compounding. The transmit voltage was lower than 20 V to guarantee the safety of patients. At least 3 acquisitions were performed in each patient at 2 weeks after decompressive craniectomy and each acquisition lasts 0.2 s.

### Signal processing

The signal processing steps of uPDI are shown in Figure 1D. The acquired ultrasound channel RF data with a size of x×z×n×t were first clutter filtered along each transmit angle using the singular value decomposition (SVD) method (Demene et al., 2015) to reject tissue signal (i.e., clutters) and preserve microvessels, where x , z , n, and t (*n* = 5, t = 200) are the numbers of axial, lateral, angular and temporal samples, respectively. The Doppler ensemble length was thus 200 frames. The three-dimensional RF data divided by transmit angle were reshaped to a two-dimensional (2D) Casorati matrix S with a size of xz×t .

The SVD of the matrix S is expressed as Equation 1:


(1) $$S=U\Delta V^{*}=\overset{t}{\underset{i=1}{\sum }}\lambda_{i}U_{i}V_{i}$$

where U and V are orthonormal matrices with sizes of xz×xz and t×t respectively, and Δ is a non-square diagonal matrix with a size of xt×t . Note that, the diagonal values of Δ are the singular values of S in a descending order and $\lambda_{i}$ is the $i^{th}$ diagonal value, where $i\in \left[1,t\right]$ . ∗ represents the transpose operation. $U_{i}$ and $V_{i}$ correspond to the spatial and temporal singular vectors of S , which are the $i^{th}$ columns of U and V .

The spatial and temporal characteristics of tissue signal, blood flow signal, and noise are different, which can be used to separate the RF data into three subspaces. Tissue signals were assumed to have higher spatial coherence than blood flow signals, while blood flow signals were considered to have higher spatial and temporal coherence than noise (Demene et al., 2015). In order to extract the blood flow signals, the singular values corresponding to tissue and noise were set to zero. The blood flow signals $S_{\mathrm{blood}}$ were reconstructed as Equation 2:


(2) $$S_{\mathrm{blood}}=\overset{l_{2}}{\underset{i=l_{1}}{\sum }}\lambda_{i}U_{i}V_{i}$$

where $l_{1}$ and $l_{2}$ are low-order and high-order singular value thresholds determined for the differentiation of tissue vs. blood flow and blood flow vs. noise. There is still no standardized approach to the problem of choosing the thresholds of the singular values marking the boundary between the tissue and the blood flow signal subspaces and the boundary between the blood flow signal and the noise subspaces, since the optimal thresholds vary dramatically with the imaging conditions. Rules of thumb for the thresholds $l_{1}$ and $l_{2}$ are the 10 and 50% of the Doppler ensemble length, respectively (Demene et al., 2015). In this study, the singular value thresholds $l_{1}$ and $l_{2}$ were fixed to 20 and 100, respectively.

Then, the reconstructed 2D blood flow data of each angle with a size of xz×t were reshaped to the original size of x×z×t . Next, the blood flow RF data were beamformed using the united spatial and angular adaptive scaling wiener postfilter based beamformer to improve the imaging quality of microvessels (Wang et al., 2023).

After that, the power Doppler images were obtained by accumulating the signal power along the temporal direction of beamformed blood flow data, which can be expressed as Equation 3:


(3) $$PD=\int |S_{\mathrm{blood}}|{}^{2}dt$$

To reduce the severe noise at far field caused by time-gain compensation (TGC) and improve the visualization of shallow microvessels, the noise equalization method was conducted to offset the depth-dependent noise (Song et al., 2017). The noise field was derived from the last order of singular values and singular vectors of the SVD filter, and the power Doppler images were normalized by this derived noise field to perform the noise equalization of different depths.

Further, to strengthen the signal from microvessels and improve the distinguishment between microvessels and background noise, a Hessian-based vessel enhancement filter was used on the power Doppler images (Frangi et al., 1998). Finally, the vessel-enhanced images were overlapped on the B-mode images for simultaneous visualization of anatomical structures and microvessels.

### Evaluation metrics

We used contrast-to-noise ratio (CNR) to quantify the results of traditional PDI and uPDI, which can be defined as follows Equation 4:


(4) $$CNR=10\times \log_{10}\left(\frac{\mu_{\mathrm{blood}}-\mu_{\mathrm{backgro}und}}{\sigma_{\mathrm{background}}}\right)$$

where $\mu_{\mathrm{blood}}$ and $\mu_{\mathrm{background}}$ represent the mean intensities of blood flow signals and background signals in the regions of interest (ROIs), respectively, and $\sigma_{\mathrm{background}}$ is the standard deviation of background signals. ROIs of blood flow signals were identified by locating the small vessels at different depths with 4 × 4 mm windows, and then the vessel boundaries in the windows were manually delineated by an experienced surgeon. ROIs of background signals were identified with 2 × 2 mm windows at the same depths as ROIs of blood flow signals. ROI areas of the blood flow signals and the background signals were similar by choosing the window sizes.

We also used vessel density to quantify the cerebral blood flow of patients at different depths. The regions with relative Doppler intensity higher than −20 dB were recognized as vessel regions empirically. Vessel density can be calculated by the ratio of the vessel region area to the total region area. Vessel density of images obtained by uPDI at the depths of 1–2 cm, 2–3 cm, 3–4 cm, and 4–5 cm were calculated for each patient.

## Results

### Patients’ information

All five patients were diagnosed with DoC, three in UWS, and two in MCS-. Specific information about the patients can be found in Table 2. The age of patients mostly ranged from 50 to 60 years, except for one pediatric patient aged 7. Their etiology includes intracerebral hemorrhage (ICH) and traumatic brain injury (TBI). The time since decompressive craniectomy ranged from 2 months to as long as 2 years.

**Table 2:** Clinical information.

N	Age	Etiology	Duration	Diagnosis	Baseline CRS-R	Outcome CRS-R	Proportion of areas with blood flow (%) at different depths			
	(y)		(d)				1-2 cm	2-3 cm	3-4 cm	4-5 cm
1	58	ICH (basal ganglia, right)	158	UWS	6 (102102)	8 (104102)	29.1	14.9	2.7	0.7
2	7	ICH (basal ganglia, right)	859	UWS	7 (112102)	8 (311102)	14.1	6.1	0.0	0.0
3	68	TBI (frontotemporal lobe, right)	81	MCS-	8 (113102)	9 (312102)	2.7	0.9	0.4	4.2
4	66	ICH (basal ganglia, left)	63	MCS-	13 (145102)	13 (145102)	13.3	6.1	1.0	0.0
5	57	TBI (subdural hematoma,right)	54	UWS	4 (002101)	4 (002101)	14.6	3.8	1.6	0.1

ICH, intracerebral hemorrhage; TBI, traumatic brain injury; MCS-, minimally conscious state minus (MCS-); UWS, unresponsive wakefulness syndrome; CRS-R, Coma Recovery Scale-Revised.

Patient 1 suffered a right-sided ICH (approximately 30 mL) in the basal ganglia, which resulted in unconsciousness. Emergency decompressive craniectomy surgery was performed to alleviate intracranial pressure. Five months later, she was admitted to our hospital for cranioplasty. Upon admission, the patient was diagnosed with UWS, with a CRS-R score of 6 (102102). Three months later, her consciousness level improved to MCS, with a CRS-R score of 8 (104102). In the ultrasound blood flow imaging, this patient exhibited the most pronounced cortical and deep-brain blood flow among all participants. Quantitative analysis of the proportion of brain regions with blood flow in the depths of 1–2 cm, 2–3 cm, 3–4 cm, and 4–5 cm showed the following values: 29.1, 14.9, 2.7, and 0.7%, respectively.

Patient 2 is a 7-year-old girl who experienced a right basal ganglia ICH that ruptured into the ventricles and underwent decompressive craniectomy 2 years ago. As an underage individual, she was ineligible to undergo cranioplasty. Her parents took her to our hospital to receive vagus nerve stimulation (VNS) to control seizures and promote recovery of consciousness. Upon admission, she was diagnosed with UWS, with a CRS-R score of 7 (112102). Three months later, her CRS-R score improved to MCS, with a score of 8 (311102), and she exhibited the ability to follow simple commands. In the ultrasound blood flow imaging, she displayed relatively rich cortical blood flow but poor deep-brain blood flow signals. Quantitative analysis of the blood flow in different depths revealed the following values: 14.1, 6.1, 0.0, and 0.0%, respectively. The lack of deep-brain blood flow may stem from severe initial damage to the deep brain nuclei, but her younger age likely enhanced her recovery potential.

Patient 3 experienced altered consciousness following a car accident 2 months prior to admission, which resulted in multiple contusions and intracerebral hemorrhage, primarily affecting the right frontal and temporal lobes. Emergency hematoma evacuation and decompressive craniectomy surgery were performed to save her life. After admission, the patient underwent cranioplasty and short-term spinal cord electrical stimulation (st-SCS) to promote consciousness recovery. Upon admission, the patient was diagnosed with MCS, with a CRS-R score of 8 (113102). Three months later, her score remained within the MCS range, with a score of 9 (312102). In the ultrasound blood flow imaging, this patient showed the poorest cerebral blood flow distribution. The blood flow proportion at different depths was as follows: 2.7, 0.9, 0.4, and 4.2%, respectively.

Patient 4 experienced a left thalamic hemorrhage, which ruptured into the ventricles, and subsequent of brain herniation 4 months prior to admission. Emergency hematoma evacuation, decompressive craniectomy, and bilateral ventriculostomy were performed. We performed cranioplasty and st-SCS to promote consciousness recovery. Upon admission, the patient was diagnosed with MCS, with a CRS-R score of 13 (145102). Three months later, his score and diagnosis remained unchanged. In the ultrasound blood flow imaging, his blood flow distribution was similar to that of Patient 2, showing relatively good cortical blood flow and lower deep-brain blood flow. The quantitative analysis of blood flow at different depths revealed the following values: 13.3, 6.1, 1.0, and 0.0%.

Patient 5 suffered from TBI resulting in subdural hematoma, and frontal and temporal lobes hematoma around 2 months prior to admission. The patient exhibited a dilated right pupil and performed emergency subdural hematoma evacuation and decompressive craniectomy. We performed st-SCS to promote consciousness recovery. Upon admission, the patient was diagnosed with UWS, with a CRS-R score of 4 (002101). Three months later, there was no change in the score or diagnosis. In the ultrasound blood flow imaging, the patient exhibited relatively good cerebral blood flow, with the quantitative analysis of blood flow at different depths showing the following values: 14.6, 3.8, 1.6, and 0.1%.

### Ultrafast power Doppler imaging

**Figure 2:**
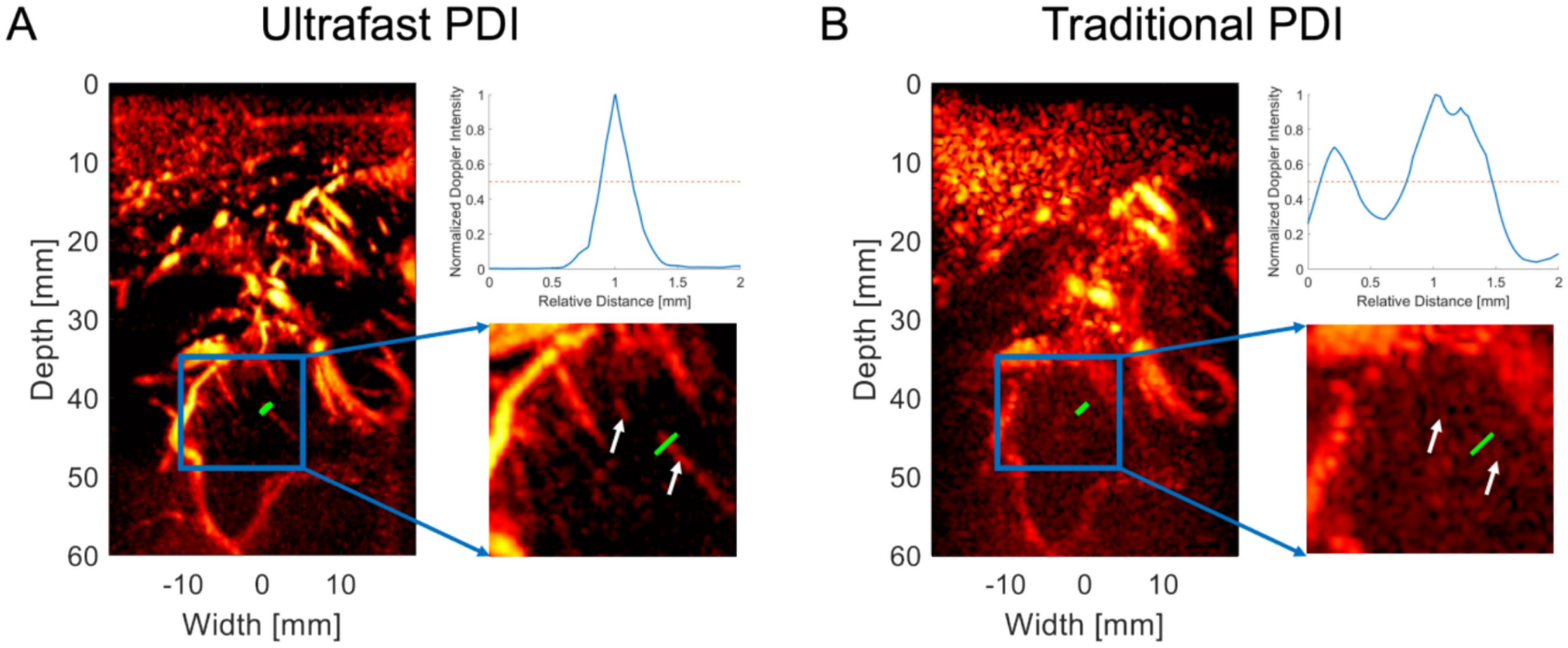
Comparative visualization of microvasculature using uPDI and traditional PDI. **(A)** Ultrafast Power Doppler Imaging (uPDI) reveals microvessels as small as 0.32 mm at 40 mm depth (green line). Fine blood vessels are resolved, as shown by the white arrows. The curve on the upper right corner demonstrates the signal intensity along the green line. **(B)** Traditional Power Doppler Imaging (PDI) before post-processing, where these microvessels are indiscernible, as indicated by the signal intensity profile along the green line on the upper right corner.

After completing various preoperative examinations and evaluations, we applied bedside uPDI to visualize the brain microvasculature through the patients’ cranial window. The proposed uPDI and the traditional PDI results for patient 1 are shown in Figures 2A,B, respectively. The signal cross-sectional profiles of the small vessel indicated by the green line in the microvascular image are shown in the upper right corners of Figures 2A,B. Zoomed-in images of the blue rectangular region in the microvascular image are shown in the lower right corners of Figures 2A,B. The normalized Doppler intensity profiles revealed that uPDI can distinguish the small vessel from the background clearly, while background speckles shows similar peaks to the signal peak from the small vessel in the traditional PDI image, which causes the confusion between the small vessel and the background speckles. The full-width at half maximum (FWHM) of the normalized Doppler intensity profile in Figure 2A was calculated as 0.32 mm, demonstrating the uPDI can visualize microvessels with diameter as low as 0.32 mm at 40 mm depth. More fine blood vessels can be resolved as indicated by the white arrows in Figure 2A while they were invisible by traditional PDI in Figure 2B.

Six groups of ROIs are identified for each patient except patient 2, whose results lack enough distinguishable vessels for quantification. Only four groups of ROIs are identified for patient 2. ROIs of blood flow signals (indicated by blue squares) and background signals (indicated by yellow squares) for patients 1–5 are shown in Supplementary Figures S1–S5. CNRs for different ROIs between traditional PDI and uPDI in the five patients are listed in Table 3. Mean and standard deviation of CNRs obtained by traditional PDI and uPDI were also calculated in each patient for comparison. A nested ANOVA was performed in Excel 2019 (Microsoft Corp., Seattle, WA) to assess the difference of the CNRs of the two methods in the four patients (1, 3, 4, and 5). The results indicate that there is a statistically significant difference in the CNRs of traditional PDI and uPDI (*p* = 1.37 × 10^−6^ < 0.001), while the difference of CNRs in different patients is not significant (*p* = 0.242 > 0.05). Therefore, compared with the traditional method, uPDI can significantly improve the CNR of microvascular images and the visibility of small vessels.

**Table 3:** Comparison of CNR for different ROIs between traditional PDI and uPDI in five patients.

	Method	ROI 1	ROI 2	ROI 3	ROI 4	ROI 5	ROI 6	Mean ± Std
Patient 1	PDI	47.10	25.35	26.30	29.27	52.99	22.10	33.85 ± 12.89
	uPDI	50.47	50.48	74.00	24.72	54.99	48.51	50.53 ± 15.75
Patient 2	PDI	−14.18	7.95	11.19	−20.08			−3.78 ± 15.66
	uPDI	16.25	20.55	43.95	15.04			23.95 ± 13.54
Patient 3	PDI	20.25	38.76	19.47	−0.81	25.06	3.38	17.69 ± 14.53
	uPDI	24.76	41.69	28.24	29.55	30.67	44.14	33.18 ± 7.84
Patient 4	PDI	−16.57	−4.66	30.84	31.25	30.73	17.76	14.89 ± 20.75
	uPDI	82.58	1.92	88.58	66.67	47.51	37.66	54.15 ± 32.23
Patient 5	PDI	−13.71	−11.87	22.58	16.49	−5.89	13.32	3.49 ± 15.81
	uPDI	104.42	51.43	64.36	73.41	22.50	54.53	61.78 ± 27.07

**Figure 3:**
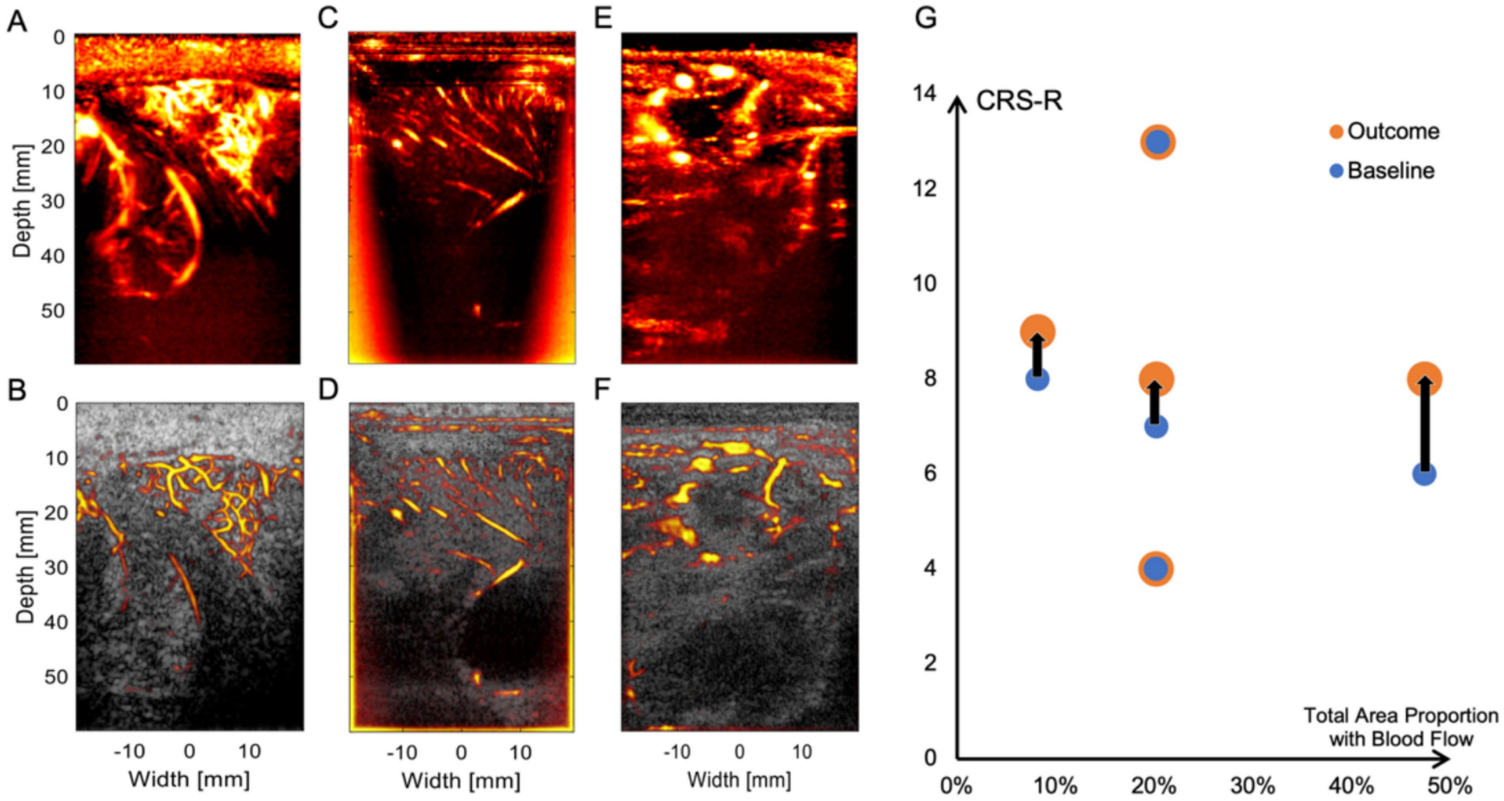
Physiological and pathological conditions represented by uPDI images. **(A,B)** Abundant cortical microvessels network of patient 5. **(C,D)** Longitudinally arranged cortical blood vessels and lateral ventricles and blood flow signals in deeper regions of patient 3. **(E,F)** Abnormal vascular cluster of patient 2. The top row displays uPDI to demonstrate the blood flow conditions, while the bottom row overlays uPDI onto ultrasound grayscale images to provide a clear visualization of the tissue structure and blood flow relationship. **(G)** Relationship between the ultrasound-scanned blood flow distribution and patients’ baseline CRS-R scores and outcomes. The black lines indicate the CRS-R score improvements of three patients (from left to right: patient 3, patient 2, and patient 1). Notably, patient 1, who exhibits the highest cerebral blood flow, shows the greatest increase in CRS-R score.

At this level of resolution, the patient’s cerebral cortical microvasculature can be clearly observed. Figures 3A,B shows a typical uPDI image of patient 5, revealing an abundant network of interconnected microvessels adjacent to the enlarged sulci. The 60 mm imaging depth through the observation window allows us not only to observe the blood vessels on the cortex of the brain but also to visualize blood flow signals from the deep brain nucleus. As shown in Figures 3C,D, uPDI could penetrate the lateral ventricles and display deep blood flow signals of patient 3 (which are crucial for prognostication of DoC). uPDI could also provide clearer visualization of certain pathological tissue. Figures 3E,F displays an area of non-perfused tissue surrounded by abnormally dilated blood vessels of patient 2, which may correspond to the high-perfusion abnormal vascular cluster in the right frontal lobe detected by Computed Tomography Angiography (CTA). Additional blood flow images of each patient can be found in the Supplementary Figure S1. We also demonstrated the capability of continuous monitoring of cerebral blood flow in patients (Supplementary Video). However, no significant changes in blood flow were observed in the imaging region during the observation period (23 s), in which the physician’s hand and the patient’s head stayed fixed as long as possible. We also noticed that any motion of the handheld ultrasound probe and the patient’s head would decrease the image quality.

## Discussion

In the present study, we were able to observe the microvascular perfusion of deep brain tissue in DoC patients with decompressive craniectomy windows using uPDI at the bedside. This technique not only has the advantages of being simple and easy to use like traditional ultrasound imaging, but it also provides microvascular visualization at sub-millimeter resolution that is comparable to contrast-enhanced CTA. Since uPDI can be performed at the bedside without the need to transport the patients to a specific location for scanning, and results can be obtained in a short time (~sec), it is highly suitable for monitoring patients’ conditions and for assessing treatment effectiveness timely.

In this study, we did not observe a clear association between the distribution of cerebral blood flow and the patients’ consciousness levels. This is likely due to the fact that consciousness involves a complex interaction between multiple brain regions, and the underlying mechanisms remain unclear. Furthermore, the blood flow distribution is heavily influenced by the type and location of the initial brain injury. In addition, vessel density may not fully reflect the blood flow distribution, as the vessel region and the background may not be optimally segmented by the pre-determined threshold. Regions with Doppler intensities lower than the threshold may not be ischemic regions. Although no statistically significant correlation was found, the patient with the most pronounced recovery exhibits the richest cerebral blood flow. That is to say, this study is only a preliminary experiment to assess the feasibility of this technique, and the small sample size prevents us from drawing statistically significant conclusions.

Regarding the quantitative analysis of uPDI, the variability in data collection sites between patients, as well as slight differences in parameters used to optimize imaging for each patient, limited its comparability across different patients. Therefore, uPDI is more suitable for intra-patient comparisons across different states rather than comparisons between patients. However, the insights gained from comparing different patients can provide some reference value. Our team is further exploring improved methods for obtaining comparable data across different patients and refining the data quantification analysis for more robust comparisons.

### Cerebral blood flow plays an important role in the diagnosis and prognosis of DoC patients

Cerebral blood flow (CBF) serves as the foundation of cerebral tissue metabolism, and the microvascular network pattern can reflect cerebral metabolism’s potentiality. The uPDI can display the cortical and deep vascular distribution of the affected hemisphere in DoC patients, thereby reflecting their metabolic potential as well. It has been found that the overall cerebral metabolism status can not only differentiate patients with different levels of consciousness (Stender et al., 2015) but also predict the outcome of DoC patients which indicated that 42% of normal cortical activity represents the minimal energetic requirement for the presence of consciousness (Stender et al., 2016). Besides, the relative metabolism changes in specific regions are associated with the preservation of specific behaviors (Yamaki et al., 2023) or sensory functions in patients with DoC (Bruno et al., 2012). Notable reductions of CBF were found in the bilateral frontal lobe, thalamus, temporal lobe, occipital lobe, and brainstem in UWS patients (Xiong et al., 2022). Most CBF or metabolism examinations require the use of contrast agents and ionizing radiation, which add burdens to the metabolic system. Magnetic Resonance Angiography is contrast-free. However, due to its time-consuming and the inability of DoC patients to cooperate (remain stable during examination), it is difficult to acquire high-quality images. Moreover, frequent visits to CT, MRI, or PET examination rooms without cardiac and respiration monitoring will increase additional risks for unstable DoC patients. These factors also limit the timeliness of the assessments and regular follow-up examinations. Although the application of intravenous microbubble-based ultrafast ultrasound localization microscopy can also characterize cerebral blood flow at the bedside (Demené et al., 2021), the intervention of contrast agents makes it difficult to perform long-term continuous monitoring. uPDI provides rich cerebral microvascular information which is closely related to brain metabolism, having great potential to effectively fulfill this demand.

### Microvascular blood flow can reflect the brain functional activity of DoC patients

There exists a close regulation between CBF and neuronal activity, known as neurovascular coupling (NVC) or functional hyperemia. Arterioles (with a caliber of between 0.3 mm and 10 μm) with smooth muscle play a crucial role in the regulation of blood flow (Gopalan and Kirk, 2022), thus important for NVC. The blood flow signals obtained by uPDI can provide fine-grained resolution down to the level of 100 μm, enabling the visualization of arterioles. As a result, it not only reflects the blood perfusion but also allows for the assessment of neuronal function, although NVC damage may occur in the traumatized brains. Applying this principle, functional near-infrared spectroscopy (fNIRS) showed that different consciousness states exhibit distinct patterns of topological architecture and connectivity within the prefrontal cortex (Liu et al., 2023). MCS patients demonstrate a significant increase in hemodynamic responses elicited by motor imagery-based tasks (Si et al., 2023). Some patients are even capable of utilizing this method to accurately answer questions and express intention (Li et al., 2021). However, due to the limited penetration capability, fNIRS can only display blood flow information up to a depth of 2–2.5 cm, thus reflecting cortical activity exclusively (Chen et al., 2020). While, for consciousness disorders, the activity of deep brain nuclei is also crucial. The widely accepted theory of the pathology of DoC is the mesocircuit hypothesis, which posits that the central thalamus is a critical node for consciousness (Schiff, 2010). Functional MRI and electrophysiology studies indicate that consciousness relies on the intricate interactions between large-scale thalamocortical and corticocortical networks (Baker et al., 2014; Redinbaugh et al., 2020). It is also reported that for Doc patients, there is a significant reduction of metabolism in the striatum, along with a relative increase in globus pallidus (Fridman et al., 2014), and there is a positive correlation between subcortical glucose metabolism and alpha power, while cortical activity shows a negative correlation with alpha power (Annen et al., 2023). Therefore, solely observing cortical activity would provide an incomplete picture. Although functional MRI enables simultaneous observation of both cortical and deep brain nuclei function and their connectivity, it is still limited by equipment and time constraints. Frequent monitoring and simultaneous implementation with various intervention methods are not available. Our study indicates that uPDI can provide clear observations of cerebral blood flow up to a depth of 6 cm in the brain tissue. Based on this technology, functional ultrasound (fUS) imaging, which is based on uPDI, holds great potential as a method that can simultaneously observe both cortical and deep nuclei activity (Mace et al., 2011).

### Continuous bedside ultrasonic monitoring of cerebral blood flow can guide neuroregulatory therapy of DoC patients

Neuromodulation therapies are substantial treatment modalities for DoC (Thibaut et al., 2019). However, in the case of DoC, there is often a lack of direct observation of clear behavioral changes or subjective improvement feedback during the parameter selection process for neuromodulation. Therefore, bedside neurophysiological and neuroimaging examinations play a crucial role in guiding neuromodulation therapies for DoC.

EEG is a widely employed technique. A study has shown that 100 Hz deep-brain stimulation can improve brain functional connectivity (Dang et al., 2023). For spinal cord stimulation therapy, we found the correlation between long-term clinical improvement and specific frequency activity in EEG (Zhuang et al., 2023) and then assessed the therapeutic effects of different parameters by examining changes in that band following treatment (Yang et al., 2022). However, the physiological significance of changes in different EEG signal characteristics is difficult to ascertain. Moreover, all conclusions require analysis over a period of time to reveal statistically significant differences within the population, making it challenging to provide timely guidance for individualized treatments.

We have also utilized fNIRS to explore the effects of different frequencies and stimulation intervals. Brief stimulation (30 s) improves cerebral blood volume in the prefrontal cortex (Zhang et al., 2018), and 70 Hz SCS enhances information transmission in the thalamocortical network pathway (Si et al., 2018). However, fNIRS can only observe changes in cortical blood flow; and changes in the deep nucleus can only be inferred without direct evidence. The breakthrough in uPDI and further fUS imaging based on this technology offer a potential solution to this problem (Mace et al., 2011). We will be able to observe the responses of deep brain nuclei and the cortex in patients under different stimulation parameters in real-time at the bedside, enabling us to promptly determine the optimal stimulation parameters tailored to each individual.

### Future potential and current limitations

uPDI is the basis of fUS. fUS can achieve functional imaging with a large field of view (on the centimeter level) with outstanding spatial resolution (around a few hundred micrometers), temporal resolution (around tens of Hz), and flow sensitivity (< 1 mm/s). It bridges the gap between functional MRI (Lee et al., 2013) and optical imaging. fUS can be operated in a noninvasive or minimally invasive manner, is user-friendly and bedside, and is relatively cost-effective compared to functional MRI. Moreover, it exhibits high compatibility with other inspection methods such as EEG. Because of these advantages, fUS has been used in many pre-clinical and clinical applications, including the imaging of brain activation maps and brain connectivity of animals (Mace et al., 2011; Osmanski et al., 2014), human newborns (Demene et al., 2017) and intraoperative patients (Imbault et al., 2017). The DoC patients naturally provide an acoustic window for the passthrough of ultrasound, allowing the implementation of uPDI and fUS. In the future, fUS will be potentially utilized to obtain the brain activation map of DoC patients, under different neuromodulation therapies such as TMS or during cognitive tasks.

However, the current uPDI technique still has some limitations for clinical implementation for DoC patients. Due to the variability of the orientation of the ultrasound probe and the severe structural damage of DoC patients, although we can visualize plenty of vessels, it is still challenging to accurately determine which specific vessels they are. Further development of a reliable localization method is necessary, which can assist the co-registration of uPDI results with other imaging modalities, like CTA or MRI. We are also working on enhancing the imaging depth and providing clearer visualization of microvessels in deep brain nuclei. In further research, we aim to observe dynamic changes in cerebral blood flow under different stimuli. Therefore, it is necessary to develop reliable spatial positioning methods for accurate measurements and image coregistrations. By overcoming these limitations, our ultimate goal is to develop a convenient and practical tool that can improve the diagnosis and treatment of DoC.

## Conclusion

The cerebral blood flow information of DoC patients is of significant value in guiding treatment decisions. However, due to the special characteristics of these patients, various examinations that require patient transfer can pose additional risks. Currently, there is a lack of methods that can display deep and high-resolution cerebral blood flow information at the bedside of the patients. In this study, we visualized the microvessels of DoC patients through the decompressive craniectomy window using the uPDI technique for the first time. Compared with conventional B-mode imaging which only provides anatomical structure information and traditional Doppler imaging with limited sensitivity, the uPDI technique can achieve functional imaging and higher sensitivity in the detection of microvessels with low-speed blood flow (~mm/s). The uPDI technique allows for real-time visualization of cerebral blood flow at the bedside without the need for contrast agents. In the future, successive uPDI monitoring will be conducted to analyze the brain function, including brain activation map and brain intrinsic connectivity, to potentially evaluate the brain function and predict the outcome of DoC patients.

## Data Availability

The raw data supporting the conclusions of this article will be made available by the authors, without undue reservation.
