# Supplementary figures and images for: Bedside cerebral microvascular imaging of patients with disorders of consciousness: a feasibility study
Source: Front Neurosci. 2025 Feb 12;19:1518023. doi: 10.3389/fnins.2025.1518023 (PMC11861210; doi:10.3389/fnins.2025.1518023)

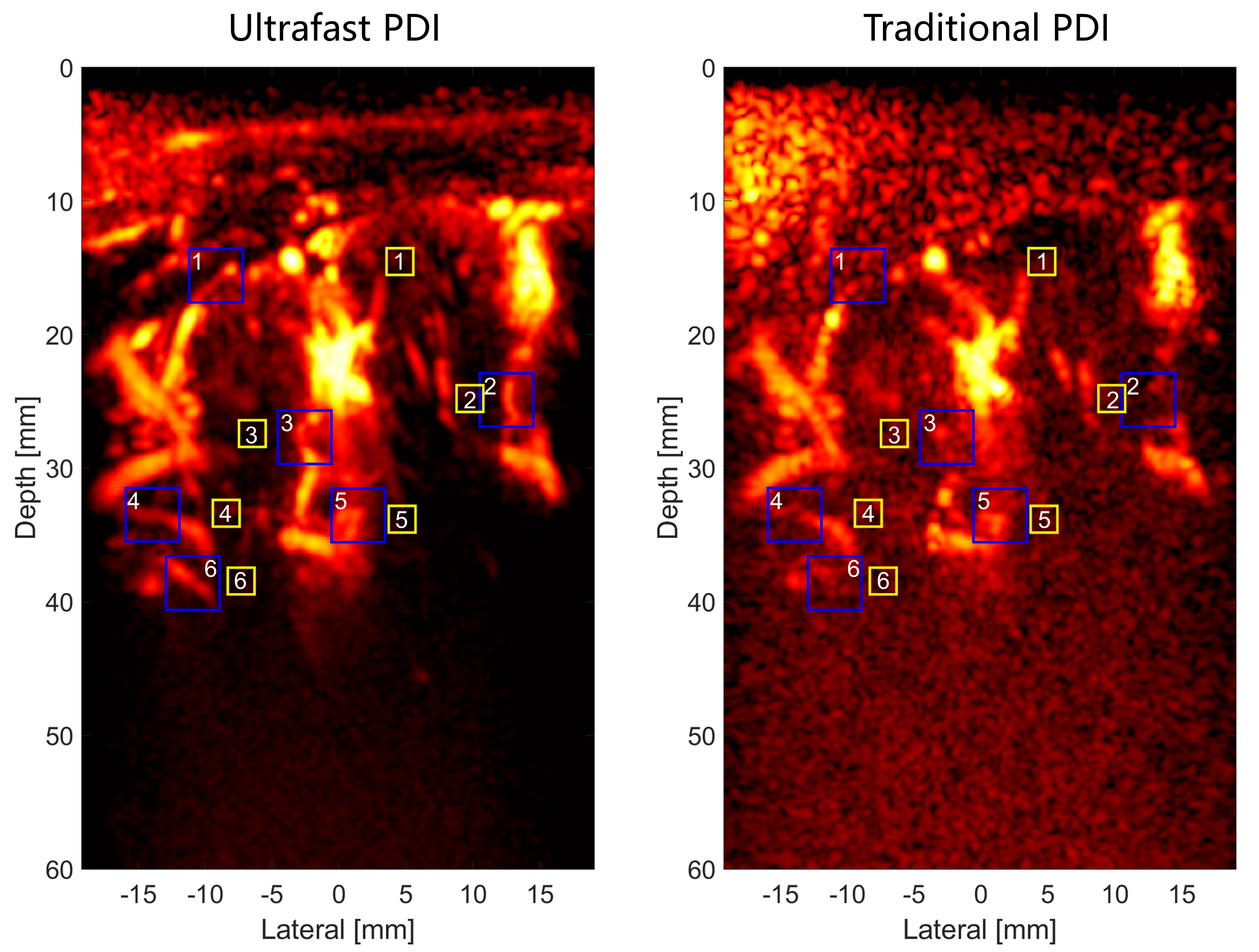

Supplement: Supplementary file 1 [file Image_1.PNG]

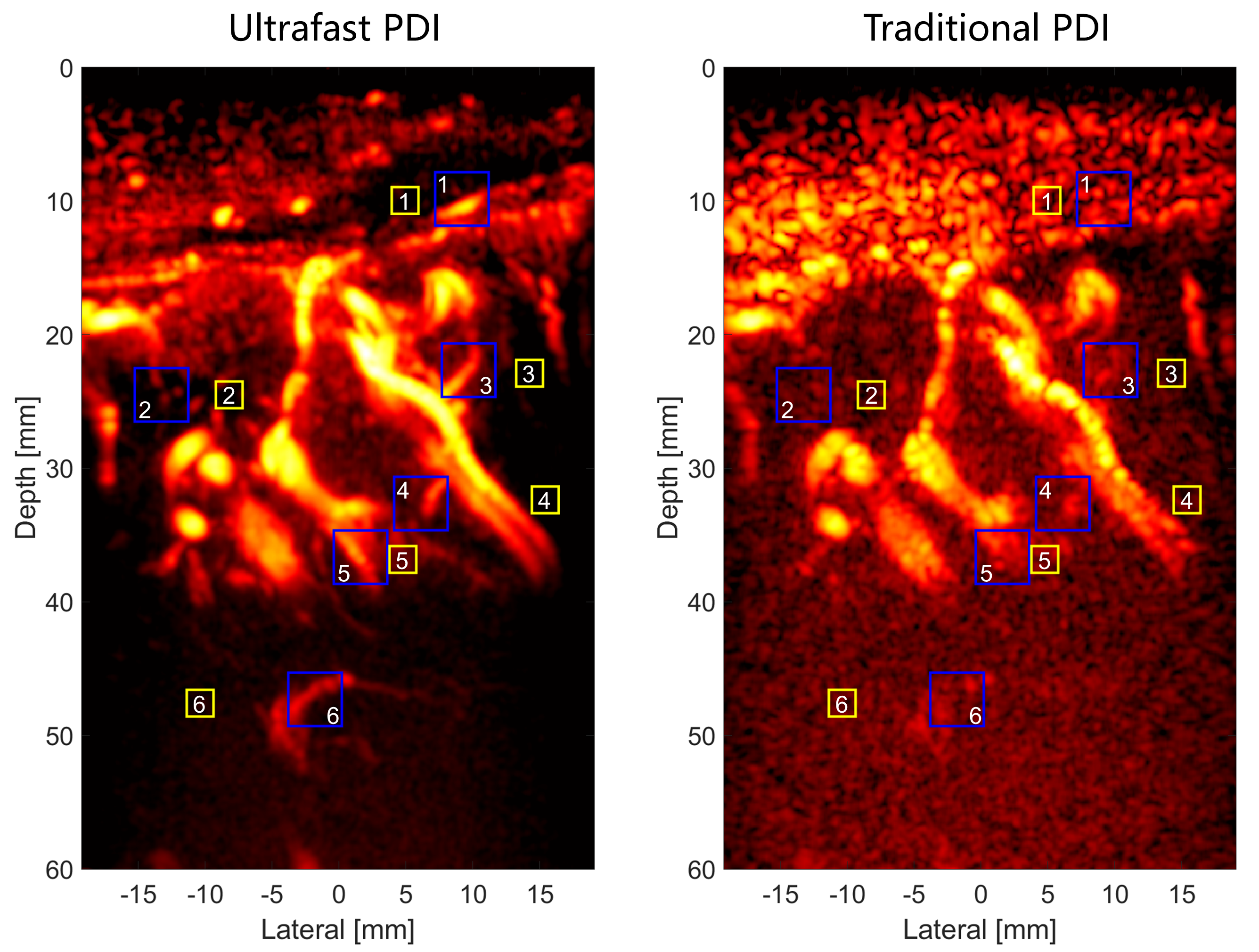

Supplement: Supplementary file 2 [file Image_2.PNG]

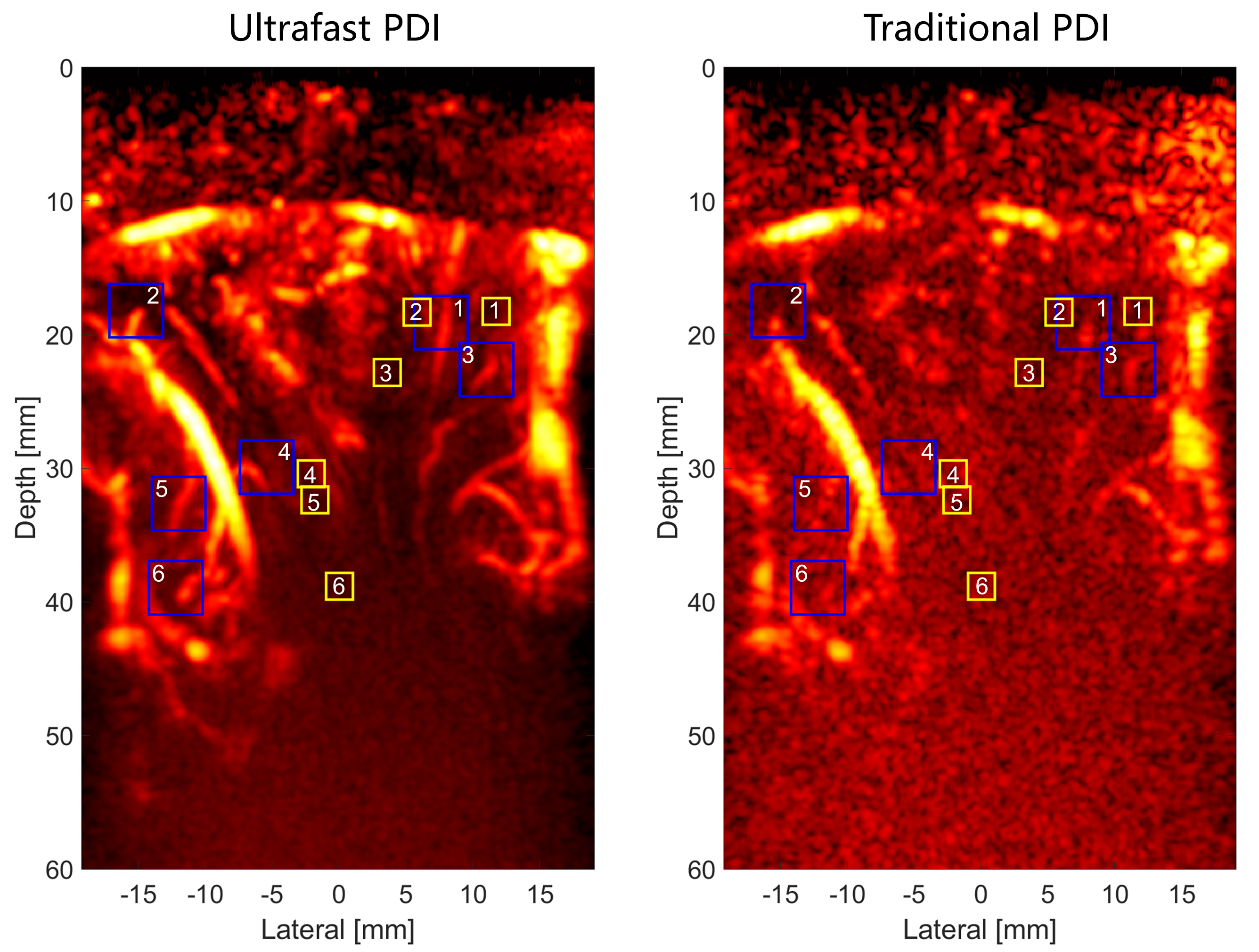

Supplement: Supplementary file 3 [file Image_3.PNG]

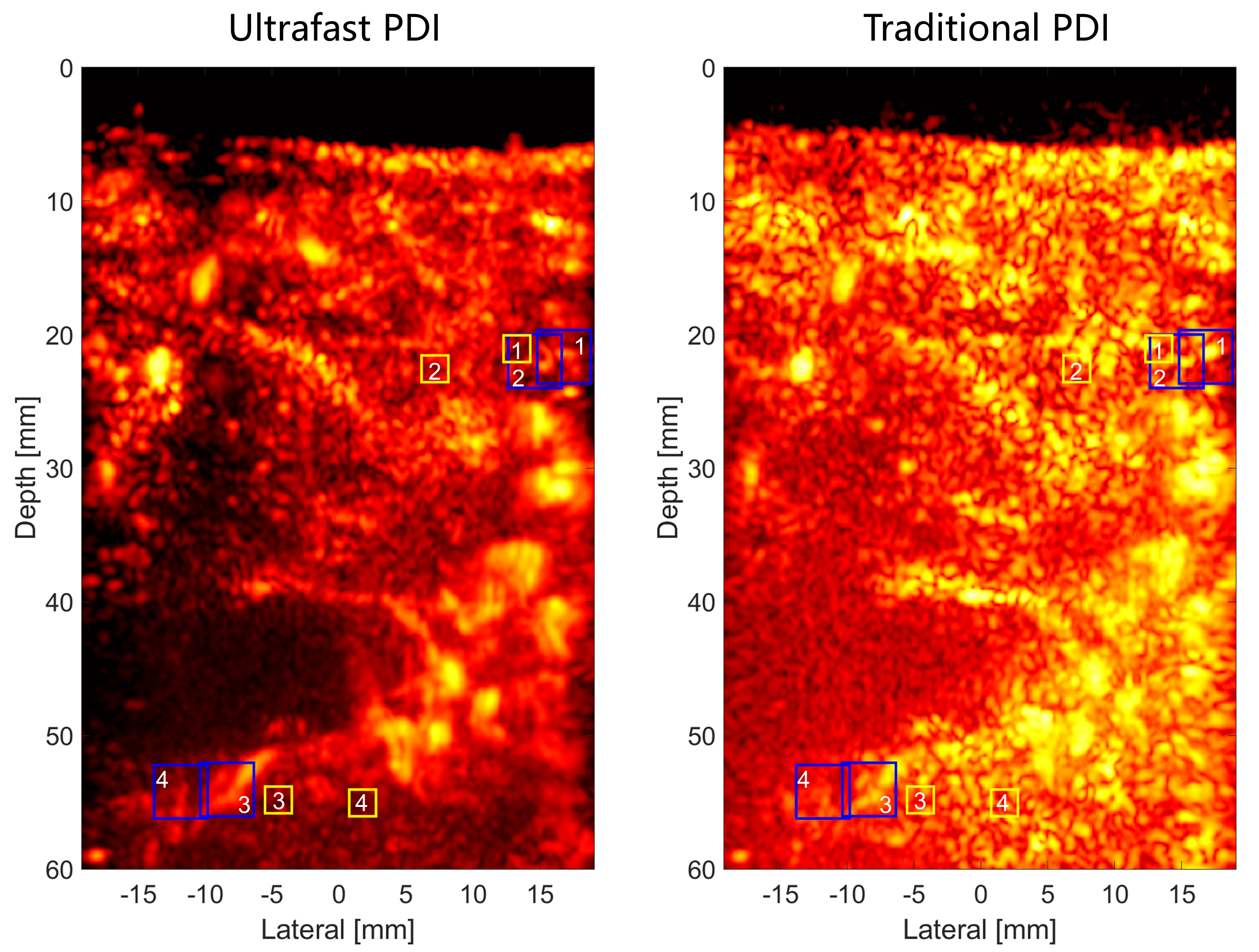

Supplement: Supplementary file 4 [file Image_4.PNG]

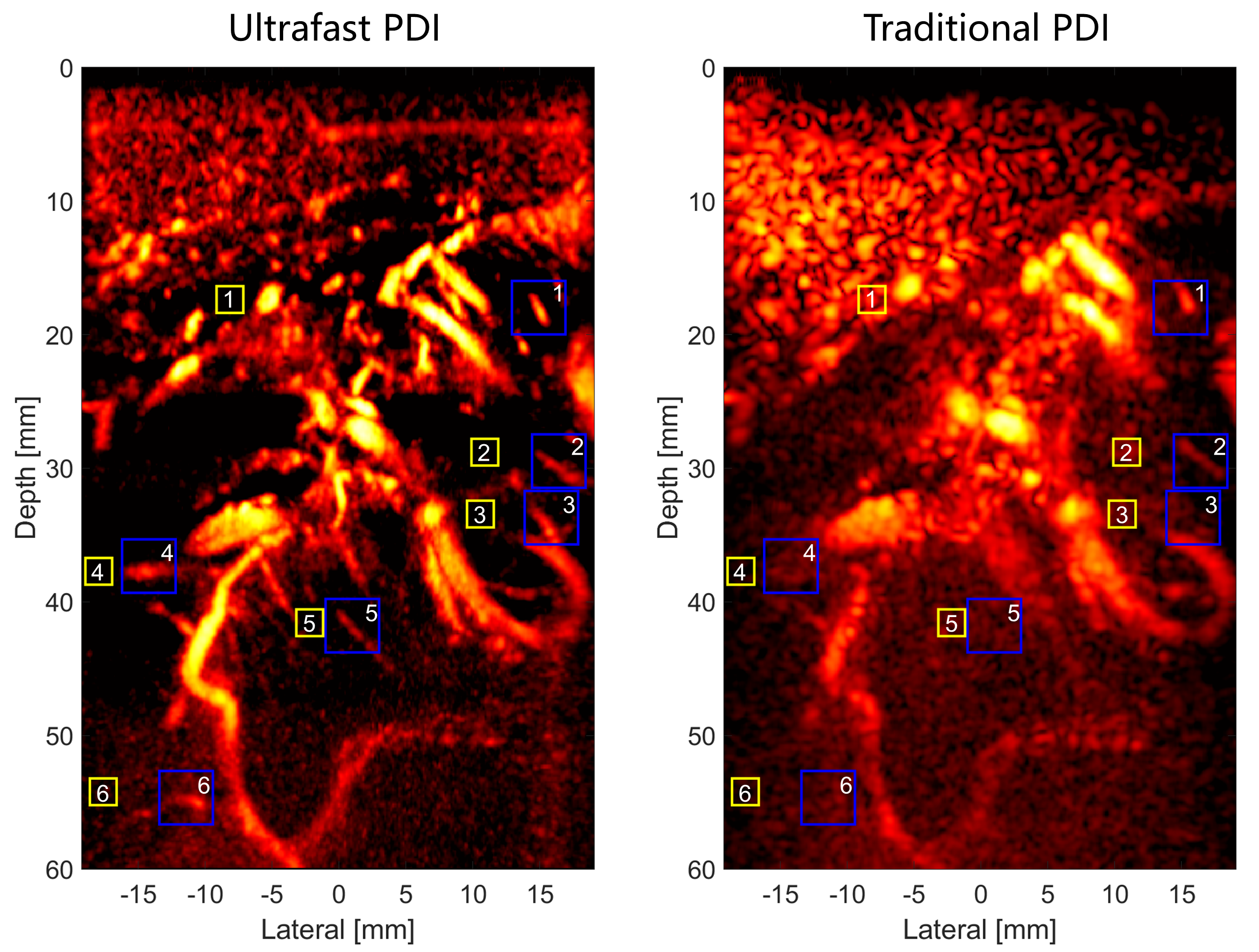

Supplement: Supplementary file 5 [file Image_5.PNG]
